# Comprehensive management of children and adolescents with type 1 diabetes mellitus through personalized physical exercise and education using an mHealth system: The Diactive-1 study protocol

**DOI:** 10.3389/fendo.2024.1354734

**Published:** 2024-02-06

**Authors:** Ignacio Hormazábal-Aguayo, Jacinto Muñoz-Pardeza, José Francisco López-Gil, Nidia Huerta-Uribe, María J. Chueca-Guindulain, Sara Berrade-Zubiri, Elisabet Burillo Sánchez, Mikel Izquierdo, Yasmin Ezzatvar, Antonio García-Hermoso

**Affiliations:** ^1^ Navarrabiomed, Hospital Universitario de Navarra (HUN), Universidad Pública de Navarra (UPNA), IdiSNA, Pamplona, Spain; ^2^ One Health Research Group, Universidad de Las Américas, Quito, Ecuador; ^3^ Pediatric Endocrinology Unit, Hospital Universitario de Navarra (HUN), IdiSNA, Pamplona, Spain; ^4^ Department of Nursing, Universitat de València, Valencia, Spain

**Keywords:** insulin-dependent diabetes mellitus, physical exercise, resistance training, mobile-health, pediatrics

## Abstract

**Introduction:**

The use of new technologies presents an opportunity to promote physical activity, especially among young people with type 1 diabetes (T1DM), who tend to be less active compared to their healthy counterparts. The aim of this study is to investigate the impact of a personalized resistance exercise program, facilitated by the Diactive-1 App, on insulin requirements among children and adolescents diagnosed with T1DM.

**Methods and analysis:**

A minimum of 52 children and adolescents aged 8-18 years, who were diagnosed with T1DM at least 6 months ago, will be randomly assigned to either a group engaging in an individualized resistance exercise program at least 3 times per week over a 24-week period or a waiting-list control group. The primary outcome will be the daily insulin dose requirement. The secondary outcomes will include glycemic control, cardiometabolic profile, body composition, vascular function, physical fitness, 24-hour movement behaviors, diet, and psychological parameters. The usability of the app will also be assessed.

**Ethics and dissemination:**

Ethical approval to conduct this study has been granted by the University Hospital of Navarra Research Board (PI_2020/140). Parents or legal guardians of minors participating in the study will provide written consent, while children and adolescents will sign an assent form to indicate their voluntary agreement. The trial’s main findings will be shared through conference presentations, peer-reviewed publications, and communication directly with participating families. This study aims to offer valuable insights into the holistic management of children and adolescents with T1DM by utilizing personalized exercise interventions through an mHealth system.

**Trial registration:**

NCT06048757

## 1 Introduction

Type 1 diabetes mellitus (T1DM) imposes a substantial burden on children and adolescents worldwide. According to estimates from the International Diabetes Federation (IDF), 1.2 million individuals under age 20 have T1DM globally (1). Not maintaining optimal glycemic control is associated with chronic health issues later in life. However, achieving and sustaining appropriate glycemic control poses a significant challenge for young individuals with T1DM, particularly during the transition from childhood to adulthood. Inadequate glycemic control in T1DM patients can lead to long-term complications including cognitive dysfunction (2), cardiovascular disease (3), diabetic neuropathy (4), diabetic retinopathy (5), chronic kidney disease (6), diabetic foot ulcers (7), and dry skin (8). While technology has enhanced self-management capacity (9), novel strategies that are accessible and cost-effective are urgently needed to improve glycemic control in young T1DM patients.

The American Diabetes Association (ADA) recommends that individuals under 20 years old with diabetes engage in 60 minutes of moderate to vigorous aerobic physical activity daily. This should be paired with vigorous muscle-strengthening and bone-strengthening activities at least three days per week (10). However, children and adolescents with T1DM are less active, more sedentary, and less fit than their healthy counterparts (11). A recent meta-analysis showed that exercise training has a moderate effect on reducing glycated hemoglobin (HbA1c) and insulin dose per day in youths with T1DM (12). Specifically, resistance training seems to be one of the most effective strategies for improving glycemic control among children and adolescents with T1DM (13). In adults, resistance exercise has proven efficacy in minimizing exercise-induced hypoglycemia risk in T1DM (14, 15). However, lack of awareness and fear of hypoglycemia can discourage young people from participating in physical activities, especially resistance exercise (16). This highlights the need for new technologies to support the T1DM population in managing hypoglycemia situations and promoting exercise.

In 2023, 6.92 billion people worldwide own a smartphone, representing 86.29% of the global population (17). Fitness apps have gained popularity among smartphone users, with some proving highly effective for increasing physical fitness (18) and physical activity levels (19). While some apps are designed for the general population and may present challenges for those with health conditions, there are also apps that are specifically helpful for managing certain conditions like T1DM. For instance, diabetes apps have shown benefits for glycemic control (20), reducing HbA1c (21), and improving health-related quality of life (HRQL) (22).

Evidence suggests that mHealth interventions can moderately reduce physical inactivity in children and adolescents (23). Specifically, a recent narrative review by Kordonouri et al. (24), explored smartphone apps for exercise management in T1DM, primarily in adults. Although emerging apps offer exercise support, none exclusively address resistance training for children and adolescents with T1DM. This gap presents a significant opportunity to improve disease management in this population. Specialized apps tailored to the unique needs of young T1DM patients could empower them to take control of their health. Such apps should consider appropriate exercise types and intensities based on fitness level. Integration with continuous glucose monitoring (CGM) systems could enable real-time feedback to prevent hypo/hyperglycemia. By promoting physical activity, enhancing fitness, and supporting effective diabetes management, these customized apps have the potential to provide significant benefits to children and adolescents with T1DM (25).

Based on prior research, our primary hypothesis posits that implementing the Diactive-1 App intervention over 24 weeks will result in a reduced daily insulin dose requirement, specifically in terms of insulin dose per kilogram of body weight, among children and adolescents with T1DM compared to standard care.

Our main aim is to compare the effects of a 24-week Diactive-1 App intervention versus standard care on insulin dose requirements in children and adolescents with T1DM. Our secondary aims are to evaluate the impact of the Diactive-1 App intervention on glycemic control, cardiometabolic profile, body composition, vascular function, physical fitness, 24-hour movement behaviors, dietary habits, and psychological well-being in comparison to the control group receiving standard care, over a 24-week intervention period.

## 2 Methods and analysis

### 2.1 Trial design

The study will be a randomized controlled single-blind parallel group study, conducted at a single center, and registered in the Clinical Trials Registry (NCT06048757). The protocol includes all elements from the Clinicaltrials.gov registry platform. This protocol is developed in accordance with the SPIRIT guidelines for randomized controlled trials (RCTs) (26).

### 2.2 Study setting

The Pediatric Endocrinology Unit at the University Hospital of Navarra, in collaboration with Navarrabiomed, located in Pamplona, Spain, is currently conducting this pragmatic trial. For additional information, please refer to the Clinical Trials Registry: NCT06048757.

### 2.3 Eligibility criteria

Children and adolescents of both sexes, aged 8-18 years diagnosed with T1DM, will be recruited as participants from the Pediatric Endocrinology Unit at University Hospital of Navarra (Pamplona, Spain). Participants will be eligible to be part of the study if they meet the following inclusion criteria: a willingness to participate in the intervention, proficiency in the Spanish language, and a minimum of six months having passed since the diagnosis. The exclusion criteria include any comorbidity that limits the capacity to participate in physical activity or an inadequate understanding of the Spanish language. Additionally, participants will be excluded if they lack an internet connection, do not own a smartphone or tablet, or do not have the ability to use the application.

### 2.4 The intervention – the Diactive-1 App

**Figure 1:**
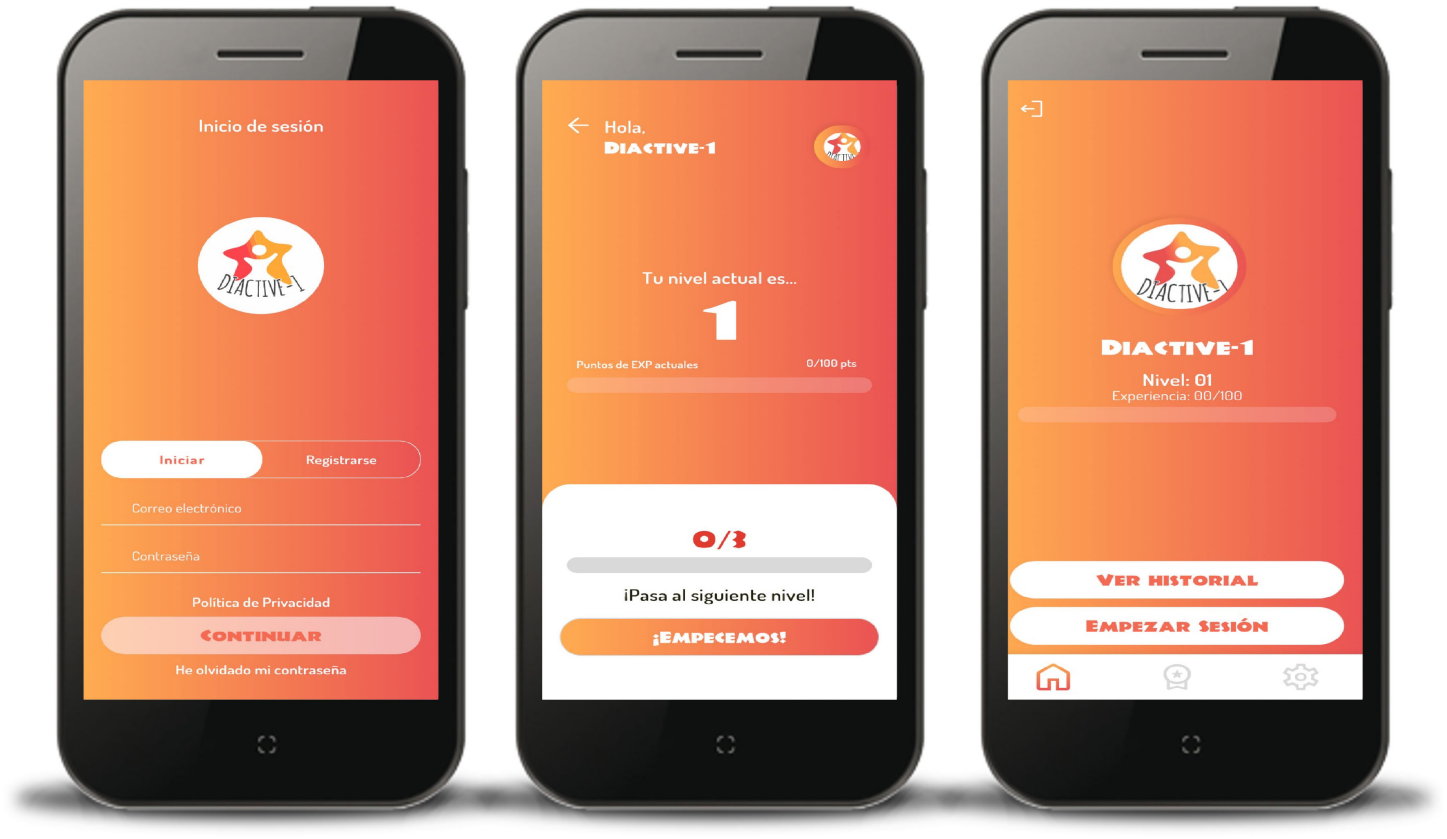
Screenshots of the Diactive-1 App for children and adolescents with type 1 diabetes.

The Diactive-1 Study is a 24 weeks smartphone intervention with the aim of improving daily insulin dose requirements, glucose control management, adherence to resistance training, and compliance with PA guidelines recommendations (10, 27, 28) for children and adolescents with T1DM. The Diactive-1 App has been developed to be compatible with both IOS and Android smartphones. A screenshot of the Diactive-1 App is displayed in **Figure 1**.

### 2.5 Development of the intervention

The Diactive-1 App was developed by a team of researchers with expertise in PA and T1DM. Intervention includes evidence-based recommendations at management of glucose control and physical exercise for children and adolescent with T1DM (10, 27, 28).

### 2.6 Content and use of the intervention

The Diactive-1 App consists of an automated program designed to offer evidence-based guidance for creating exercise training sessions (10, 27, 28). The sessions are tailored based on the individual’s physical fitness level (assessed beforehand), glucose levels, and glucose trend arrow at the moment. Furthermore, the Diactive-1 App can be integrated with CGM Freestyle 2 devices to display glucose levels and trend arrows prior to the commencement and at the end of the session. If patients use a different device for CGM (e.g., MiniMedTM 780G, Medtronic), the application will ask them to manually enter their glucose levels and trends before and after the exercise session.

Educational glucose monitoring is a feature included in the Diactive-1 App. This feature presents messages after users input their glucose levels and trend arrow. Depending on these two parameters and, in special cases, patients may also be prompted to input their ketone levels. In accordance with the position statement from the European Association for the Study of Diabetes (EASD) and the International Society for Pediatric and Adolescent Diabetes (ISPAD) (27), the Diactive-1 app sends advisory messages to the patient based on their present condition. For instance, if their blood glucose exceeds 330 mg/dL regardless of ketone levels, the app recommends refraining from exercise, suggests correcting glucose levels with insulin, and proposes attempting exercise again after a 30-minute interval.

The Diactive-1 App incorporates a gamification concept. Each patient starts at player level 1, which increases based on the number of training sessions completed. As previously mentioned, each patient must complete three sessions per week (mandatory) with a maximum of seven sessions (including the three mandatory ones). Completing each of the mandatory sessions’ rewards participants with 20 experience points. Additional sessions beyond the mandatory three yield 30 experience points each. Upon accumulating 100 experience points, they advance to the next level. To continue leveling up, they must accumulate another 100 experience points. Furthermore, the Diactive-1 App includes a ranking system that positions patients based on their player levels. This feature aims to encourage healthy competition and promote patient adherence to using the Diactive-1 App.

**Figure 2:**
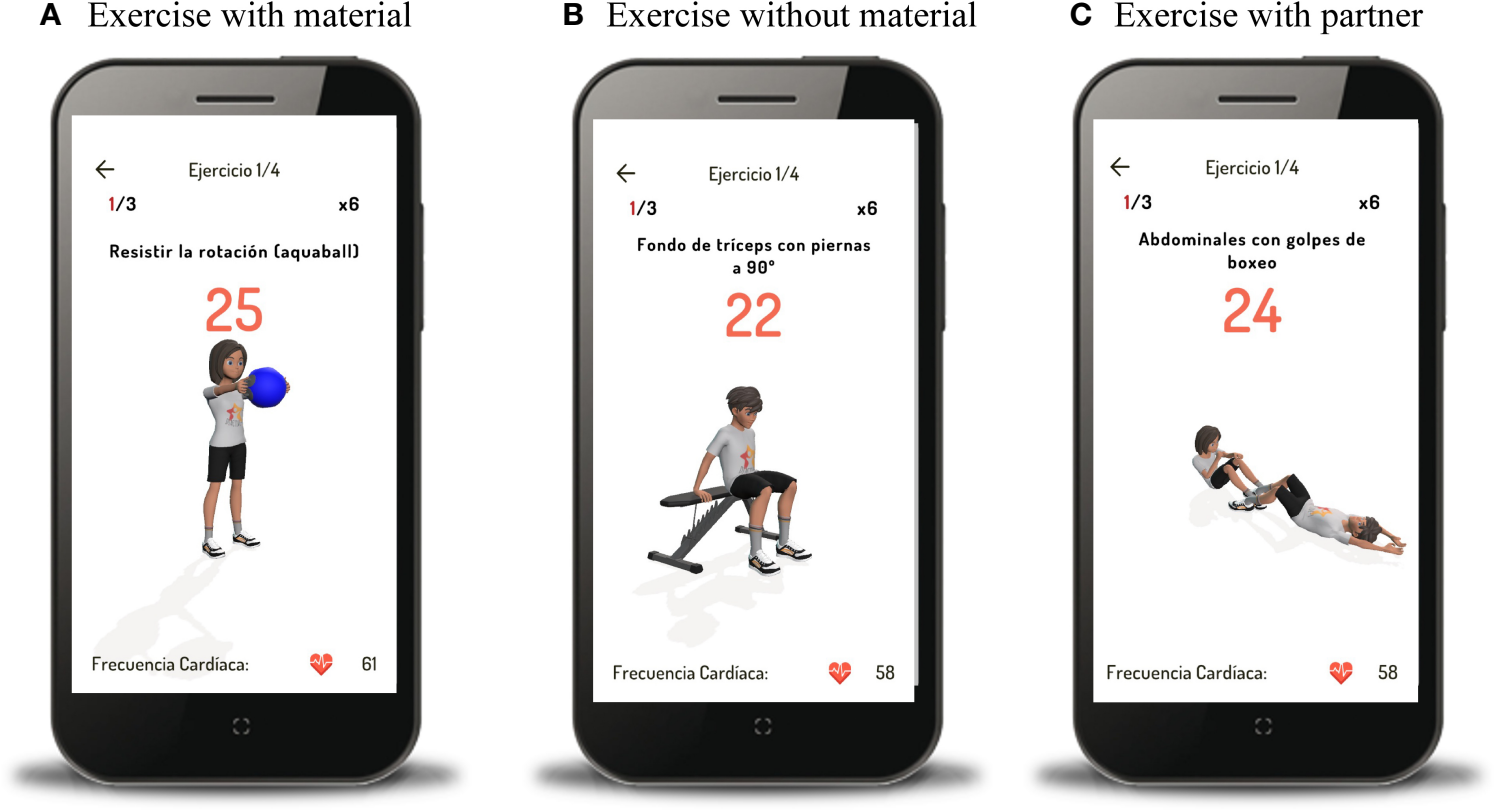
Example of guided exercises with material **(A)**, without material **(B)**, and with partner **(C)**.

The interventions offered by the Diactive-1 App can take place at locations chosen by the participants, such as their homes, parks, or schools. A face-to-face session will be conducted before commencing the intervention to ensure that participants are familiar with the fundamental movements, thus reducing the risk of potential muscle injuries. Each training session is designed to last between 13 and 33 minutes. The level of fitness—low, medium, or high—determines the number of exercises within the sessions: four exercises for those with low fitness and five for those with medium and high fitness levels. Reference values for handgrip strength in European children and adolescents aged 6 to 18 will be employed (29). Using the median percentile of the handgrip strength, individuals falling below the 20th percentile will be classified as having low fitness levels, those between the 21st and 79th percentiles will be considered to have moderate fitness levels, and those at or above the 80th percentile will be categorized as having high fitness levels, with consideration for their sex and age. The training sessions consist of three types: equipment-based training (utilizing resistance bands and an aqua ball, which will be provided to the participants before the intervention), equipment-free training (bodyweight exercises), and partner-assisted training. Participants will be guided through their workouts by a 3D Avatar displayed on their smartphone screen. This Avatar will show the exercises for the training session, offering visual cues for correct movements (**Figure 2**). Additionally, a background voice (narrator) will provide verbal instructions, including the start and end of each set, transitions to the other side (in the case of unilateral exercises), and designated rest periods.

The Diactive-1 App’s training program will include performing 3 to 4 sets, each comprising 6 to 12 repetitions. When training with equipment, the weight level of the aquaball and the resistance color band will vary based on the participant’s fitness level and age group: 8-12 for children and ≥13 for adolescents. However, training without equipment will follow the same prescribed regimen for both children and adolescents. Progression in weight level (aquaball) is calculated based on the average body weight of the longitudinal Diactive-1 project participants (approximately 60 kg). This progression is tailored to specific body segments (upper limb, lower limb, and core) and fitness levels. The exercises are categorized into three main muscle groups: upper body, lower body, and core. Examples of exercises include bench press, triceps press, squat, leg extensions, plank, and sit-ups. In exceptional cases, if the Diactive-1 App determines that performing resistance training would be counterproductive (for instance, when glucose levels range from 271 to 330 mg/dL with a trend arrow indicating an increase, diagonal increase, or to the right, and ketone levels are ≤ 1.5 mM before the exercise session), the session will exclusively consist of aerobic exercise. In such situations, the patient can return to the app and proceed with their strength training session later, provided their blood glucose levels allow it. Recovery periods between sets will range from 30 to 60 seconds, while recovery between exercises will last from 60 to 75 seconds. After completing five training sessions, the training load will increase. This can involve adding more repetitions, sets, kilograms of weight (if using an aquaball), or using a different color band (if using an elastic band). Users can access these options within the Diactive-1 App. Various progression examples are included in **Supplementary Tables 1**-**10**. After participating in the intervention for four and a half months, participants will transition from their current fitness level to the next tier (e.g., from low to medium or medium to high fitness), receiving new intensities and exercises accordingly. The overall program will span 24-weeks, equivalent to a minimum of 72 sessions.

The waiting-list control group will receive standard hospital care, and after 24 weeks of the Diactive-1 App intervention, they will be given access to the App and training material.

**Figure 3:**
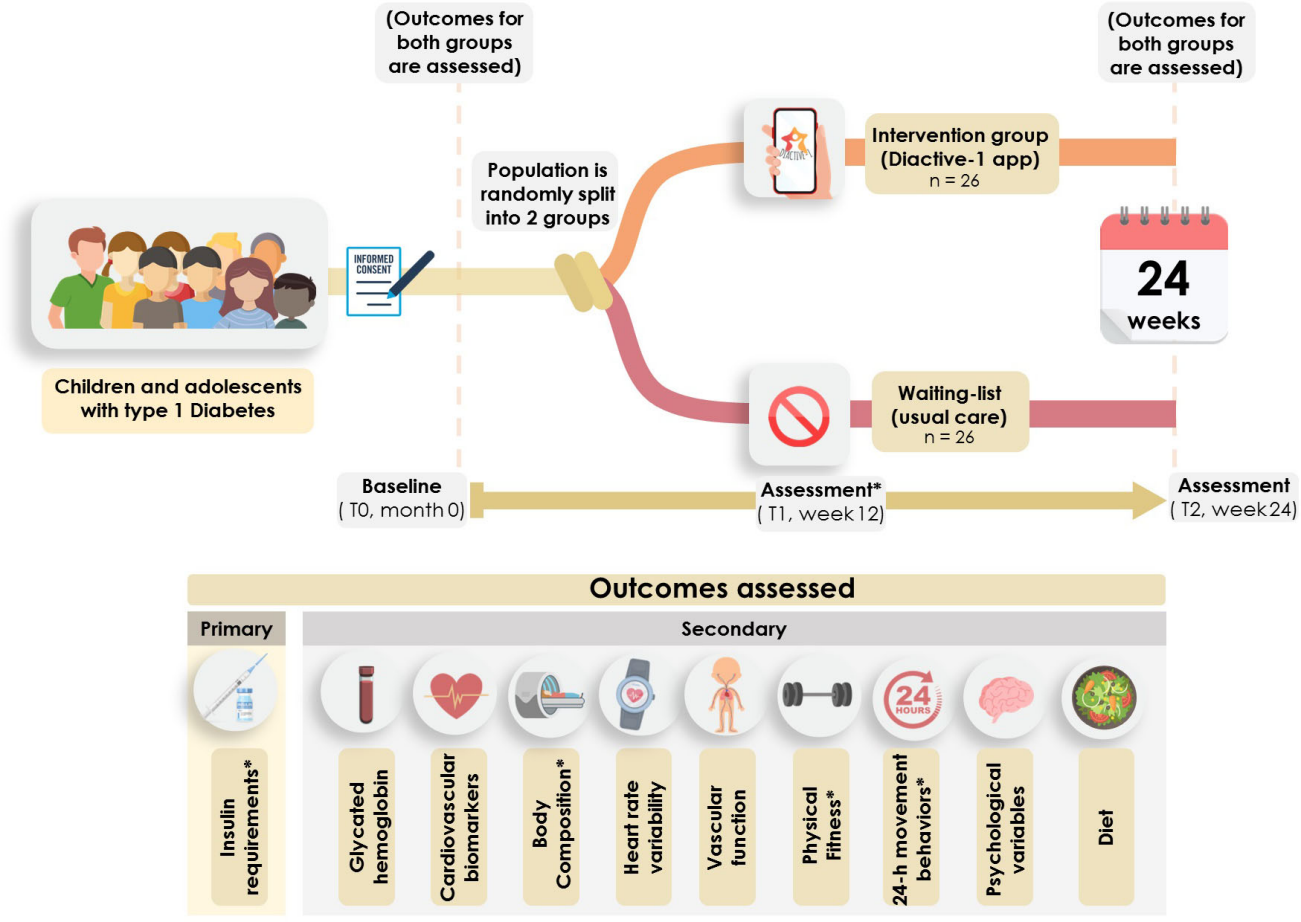
General procedure and timeline of the Diactive-1 study. * Insulin dosage assessments, body composition measurements, handgrip strength evaluations, and accelerometer-based physical activity assessments will be conducted at baseline, 12 weeks, and 24 weeks.

An overview of the general procedure is provided in **Figure 3**.

### 2.7 Intervention adherence measurement

An electronic monitoring system will be used to oversee participants’ adherence to the training sessions. This will involve checking our database to verify the sessions completed by the participants. Additionally, we will employ the Polar Varity Sense device (Polar Electro, Kempele, Finland), which displays heart rate (HR) during training sessions, to facilitate verification. Throughout the intervention, the research team will maintain regular communication with participants through phone calls and messages to provide encouragement and support, with the aim of promoting participant retention. Moreover, participants will receive a monthly report detailing goal achievement summaries and their progress relative to other participants.

### 2.8 Outcomes

A summary of the variables analyzed in the Diactive-1 Study is presented in **Table 1**.

**Table 1:** Summary of the variables examined in the Diactive-1 App study.

Outcome	Measurement	Tool
Insulin dose requirements	Self-reported/Objective	*Ad hoc* diary/Insulin pump
Glycemic control	Objective	FreeStyle 2® or MiniMed™ 780G
Cardiovascular biomarkers	Objective	Central laboratory of the University Hospital of Navarra in Pamplona, Spain
Anthropometric	Objective	SECA 213 stadiometer and SECA electronic scale (Scale 869)
Sexual maturation	Objective	Tanner criteria
Peak height velocity	Objective	Moore’s equations
Body composition	Objective	DXA Lunar iDXA, GE Healthcare
Heart rate variability	Objective	Polar V800
Vascular function	Objective	Vasera VS-2000 Vascular Screening System
Cardiorespiratory fitness	Objective	Cosmed K5 b2
Muscular fitness	Objective	Takei III Smedley Type Digital Dynamometer and EGYM Smart Strength machines
Physical activity, sedentary time, and sleep duration (accelerometers)	Objective	GENEActive triaxial accelerometer (ActivInsights)
Sleep quality and duration	Self-reported	PSQI
Sedentary behaviors (screen time)	Self-reported	YLSBQ
Self-reported physical activity	Self-reported	*Ad hoc* questionnaire
Self-reported physical fitness	Self-reported	IFIS
Inadvertent hypoglycemia	Self-reported	Clarke test
Sociodemographic information	Parent-reported	*Ad hoc* questionnaire.
Health-related Quality of life related to chronic diseases (T1DM)	Self-reported	Disabkids
Health-related Quality of life	Self-reported	KIDSCREEN-10
Subjective well-being	Self-reported	CUBE
Adherence to the Mediterranean diet	Self-reported	KIDMED
Food consumption	Self-reported	FFQ
Disordered eating	Self-reported	mSCOFF
Usability of the app	Self-reported	uMARS
Adherence with intervention (engagement)	Objective	Diactive-1 App

CUBE, Cuestionario Único de Bienestar Escolar; Disabkids, Questionnaire for Young people with diabetes; IFIS, The International Fitness Scale; KIDSCREEN, Screening for and Promotion of Health Related Quality of Life in Children and Adolescents; KIDMED, Mediterranean Diet Quality Index for Children and Teenagers; mSCOFF, Modified SCOFF questionnaire; uMARS, User Version of the Mobile Application Rating Scale; PSQI, The Pittsburgh Sleep Quality Index; YLSBQ, Youth Leisure-Time Sedentary Behavior Questionnaire.

### 2.9 Primary outcome

The primary outcome measure is the daily insulin dosage requirement. Participants will maintain a diary for nine days, recording their carbohydrate intake and insulin doses. This diary will record information about insulin injections. Children and adolescents who use an insulin pump will be assessed by obtaining objective information through downloading their data. The collected information will be used to calculate the insulin units per day per kilogram of body weight. A comparison will be made by providing participants with the same diary again, both at 12 weeks into the intervention and 9 days before the intervention concludes at week 24.

### 2.10 Secondary outcomes

#### 2.10.1 Glycemic control

A significant portion of the sample will use either the CGM FreeStyle 2® Libre device (Abbott Diabetes Care) or the MiniMedTM 780G (Medtronic) during the intervention period. These devices measure interstitial glucose levels every 60 seconds and generate glucose values every 15 minutes, along with corresponding glucose curves. The collected data will be summarized in the ambulatory glucose profile report, including the following percentages of time-in-range (TR) (30): very high (glucose >250 mg/dL), high (181–250 mg/dL), target (70–180 mg/dL), low (54–69 mg/dL), and very low (<54 mg/dL). Additionally, the glucose coefficient of variation (CV) will be calculated (30) and the number of hypoglycemic events per day, mean glucose level during this period, and percentage of time the CGM sensor was active will be recorded. In accordance with ADA guidelines (10), we will consider the following metrics as meeting glycemic targets: HbA1c <7%; CV ≤36%; TR very high <5%; TR high <25%; TR target >70%; TR low <4%; and TR very low <1%.

### 2.11 Cardiovascular biomarkers

Venous blood samples will be collected from the antecubital vein between 7:00 and 9:00 AM after a 10–12 hour overnight fast. These samples will encompass measurements of fasting glucose, glycated hemoglobin, triglycerides, high-density lipoprotein cholesterol, low-density lipoprotein cholesterol, apolipoproteins, alanine aminotransferase (ALT), and aspartate aminotransferase (AST). All assessments will be conducted both before and after the intervention at the central laboratory of the University Hospital of Navarra in Pamplona, Spain.

### 2.12 Anthropometric parameters

Standing height will be measured in bare feet using a SECA 213 stadiometer (Hamburg, Germany). Participants will be instructed to stand with their heels together and touching the base of the vertical measuring column, with their back straight and their head positioned in the Frankfurt horizontal plane (31). The standing height will be recorded to the nearest 0.1 cm.

Sitting height will be measured using the SECA 213 stadiometer and a wooden box.

Body weight will also be measured in bare feet and light clothing, using a SECA electronic scale (Scale 869), and recorded to the nearest 0.1 kg. Body mass index (BMI) will be calculated by dividing the weight in kilograms by the square of height in meters.

### 2.13 Sexual maturation and peak height velocity

Sexual maturation will be assessed by the Pediatric Endocrinology Unit at the University Hospital of Navarra (Pamplona, Spain), reporting the pubertal status on a scale of 1 to 5 in relation to secondary sexual characteristics. The assessment will be conducted using the Tanner and Whitehouse criteria (32). For girls, assessment will be based on the stage of breast development (Tanner A) and the distribution of pubic hair (Tanner B), while for boys, assessment will be based on the stage of genital development (penis size and testicular volume - Tanner A) and pubic hair distribution (Tanner B).

To obtain the peak height velocity (PHV), a common indicator of growth and development in children and adolescents (33), we will use anthropometric measures (weight, height, and seated height) as per Moore’s equations (34). To calculate the years after PHV, we will subtract the age at PHV from the actual age. The difference in years between these values will be referred to as the maturity offset.

### 2.14 Body composition parameters

Total body fat, lean mass, subcutaneous and visceral adiposity, bone mineral content and density will be measured using dual-energy x-ray absorptiometry (DXA Lunar iDXA, GE Healthcare). Participants will be positioned in a supine position, with their arms slightly separated from the body and their feet and legs hip-width apart. This assessment will take place in weeks 12 and 24.

### 2.15 Heart rate variability

Heart rate variability (HRV), sympathetic and parasympathetic nervous system indices, as well as low and high frequencies, will be measured using the Polar V800, among other variability data. These measurements are related to autonomic function at the cardiac level and serve as indicators of autonomic dysfunction at this level. The HRV data will be analyzed using Kubios software (Kubios HRV Premium, ver. 3.5, Kubios Oy, Kuopio, Finland) (35).

### 2.16 Vascular function

Vascular function will be measured in the four extremities, the cardio-ankle vascular index, the brachial-ankle pulse wave velocity and the ankle-brachial index at rest using the Vasera VS-2000 Vascular Screening System (Fukuda Denshi, Japan).

### 2.17 Physical fitness components

Cardiorespiratory fitness will be assessed through a graded stress test using ergospirometry (Cosmed, K5 b2, Italy) on a cycle ergometer (Excalibur Sport 925909, Lode, The Netherlands). A standardized protocol for children and adolescents will be followed, including a warm-up phase, a systematic increase in resistance (10 or 20W per minute) until reaching maximum exertion, and then transitioning into the recovery phase. Peak oxygen consumption (VO_2peak_) and metabolic equivalents (METs) will be determined.

Muscular fitness will be measured by handgrip strength using the Takei III Smedley Type Digital Dynamometer, which provides an estimate of an individual’s overall strength. Then, EGYM Smart Strength machines (developed by eGym® GmbH in Munich, Germany) will be used to measure both maximal strength and muscular power in upper (chest and arms) and lower (legs and hip) extremity muscles. Handgrip strength assessment will take place in weeks 12 and 24.

### 2.18 Physical activity, sedentary time, and sleep duration by accelerometers

The volume and intensity of physical activity will be measured using a GENEActive triaxial accelerometer (ActivInsights) worn on the wrist of the nondominant hand. The accelerometers will be programmed to measure at a frequency of 87.5 Hz over a period of nine consecutive days (36). The research team will determine that sampling 86 times per second is sufficient to capture the majority of movements performed by patients. The accelerometer data will be extracted using GENEActiv PC Software (version 3.3) and processed and analyzed using the R package GGIR (37). Waking wear time for valid cases represented children and adolescents with at least seven days and at least 10 hours of waking wear time in a 24-hour period, including one weekend day, will be considered for analysis. Validated cut points will be used to determine different physical activity variables (38, 39): sedentary activity (for children: 0–56.3 mg; for adolescents: 0–50 mg), light physical activity (for children: 56.3–191.6 mg; for adolescents: 50–150 mg), moderate physical activity (for children: 191.6–695.8 mg; for adolescents: 150–500 mg), and vigorous physical activity (for children: >695.8 mg; for adolescents: >500 mg). Moderate-to-vigorous physical activity will be defined as activities for which at least 80% of 1 minute of time satisfies the moderate physical activity threshold criteria (i.e., 191.6 mg for children and 150 mg for adolescents), in order to remove signals related to random wrist movement (40). The duration of sleep will also be determined. According to van Hees et al. (41) a sleep algorithm will be used to detect sleep and wake between bedtime and get uptime.

### 2.19 Sleep quality and duration

The Pittsburgh Sleep Quality Index (PSQI) will be used. It evaluates seven established aspects of sleep quality: subjective sleep quality, time taken to fall asleep, duration of sleep, sleep efficiency, sleep disturbances (such as nightmares, pain, or feeling too hot or cold), use of sleep medication, and daytime dysfunction (42).

### 2.20 Sedentary behaviors (screen time)

Sedentary behaviors will be assessed using the Youth Leisure-Time Sedentary Behavior Questionnaire (YLSBQ). Participants will report the time spent on TV, video games, computers, and mobile phones on both weekdays and weekends. To calculate the weighted average daily sedentary screen time for each behavior, we will use a 5:2 ratio. For instance, this calculation involves multiplying the daily TV viewing time on weekdays by five, the daily TV viewing time on weekend days by two, and then dividing the sum by seven (43). The total daily sedentary screen time will be determined by summing the durations of various daily screen time activities. Additionally, total screen time for both weekdays and weekends will be calculated.

### 2.21 Self-reported physical activity

The measurement of physical activity will be based on the following question: “Typically, how many days will you engage in physical activity for a total of at least 60 minutes?”. Response options will range from 0 to 7 days per week, in 1-day increments. Physical activity will be defined as less than 60 minutes of physical activity per day on at least 7 days per week (44). The measurement of muscle-strengthening activities will be based on the following questions: “In the past 7 days, how many days did you perform exercises to enhance or tone your muscles, such as pushups, sit-ups, or weightlifting?” The response choices ranged from 0 to 7 days.

### 2.22 24-h hour movement guidelines

Participants who engaged in at least 60 minutes of moderate to vigorous physical activity per day and at least three days of muscle-strengthening activities, had less than two hours of recreational screen time per day, and achieved uninterrupted sleep for 9 to 11 hours per day (for children) or 8 to 10 hours per day (for adolescents) will be categorized as meeting the comprehensive 24-hour movement guidelines (45).

### 2.23 Self-reported physical fitness

The International Fitness Scale (IFIS) is designed for assessing self-reported physical fitness. This scale consists of five elements that employ a 5-point Likert scale to inquire about the children’s general perception of their physical fitness, as well as their perception of their cardiorespiratory fitness, muscular fitness, speed-agility, and flexibility relative to their peers. The Likert scale provides choices ranging from very poor to poor, average, good, and very good physical fitness (46, 47).

### 2.24 Inadvertent hypoglycemia

The perception of hypoglycemia will be measured using the Clarke test, which consists of eight questions with different possible answers, A score greater than three reflects impaired awareness of hypoglycemia (48).

### 2.25 Sociodemographic information

Self-reported variables will be collected via a questionnaire administered to the participants’ parent(s) or guardian(s). The questionnaire will cover participant details such as school, sex, age, birthplace, race/ethnicity, and language spoken at home. Additionally, it will include information about the parent(s) or guardian(s), including birthplace, age, education level, professional qualifications, employment status, job role, monthly income, household location, neighborhood, and birth weight.

### 2.26 Psychological assessments

To evaluate the HRQL in context of a chronic illness, the Spanish version of the “Questionnaire for Young people with Diabetes” (DISABKIDS) will be used (49). This questionnaire comprises 12 questions about how a patient has felt in the last four weeks that require answers on a 5-point Likert scale from 1 (never) to 5 (always).

We will also assess the HRQL using the Screening for and Promotion of HRQL in Children and Adolescents (KIDSCREEN-10) (50). This is a generic 10-item unidimensional instrument that focuses on the functional, mental, and social aspects of well-being in children and adolescents aged 8-18 years. The instrument will consist of the following items, starting with “thinking of last week, have you: 1) felt physically fit and well, 2) felt full of energy, 3) felt sad, 4) felt lonely, 5) had enough time for yourself, 6) been able to do the things you want in your free time, 7) felt treated fairly by your parent(s), 8) had fun with your friends, 9) got along well at school, 10) been able to pay attention at school?”. For each item, participants will provide their responses on a five-point scale, ranging from “never” to “always” or from “not at all” to “extremely”.

We will also assess subjective well-being using the “*Cuestionario Unico de Bienestar Escolar*” (CUBE) (51). The CUBE questionnaire consists of 5 items that assess various aspects of life satisfaction. All these variables will be measured using a 10-point Likert scale ranging from 0 to 10 (0 = totally disagree, 10 = totally agree).

In terms of positive affect, the scale includes five items assessing emotions such as happiness, joy, cheerfulness, contentment, and fun. Additionally, there are five items evaluating negative affect, which encompasses feelings of humiliation, annoyance, irritation, bitterness, and sadness. The scale follows a bifactorial structure with five items per factor.

### 2.27 Adherence to the Mediterranean diet

We will use the Mediterranean Diet Quality Index for Children and Teenagers (KIDMED) index to assess adherence to the Mediterranean diet (52). The KIDMED index ranges from 0 to 12 and is based on a 16-question test. Unhealthy characteristics associated with the Mediterranean diet are assigned a score of -1 point, while healthy characteristics receive a score of +1 point.

The sum of all scores obtained from the KIDMED test will be utilized to classify individuals into three different levels: (a) optimal Mediterranean diet (>8 points), (b) improvement needed to align with Mediterranean dietary patterns (scores ranging from 4 to 7), and (c) very low diet quality (≤3 points).

### 2.28 Food consumption

Food frequency consumption will be assessed some food frequency questionnaires (FFQs) for the Spanish young population (53, 54). These FFQs includes several items groped into 17 food groups and were previously validated for its use among children (54) and adolescents (53) in a self-reported way. Subsequently, macronutrients, micronutrients, total energy consumption and other diet-related variables will be estimated. On the other hand, adherence to the healthy and sustainable dietary recommendations (e.g., fruits, vegetables, nuts, etc.) of the Ministry of Consumer Affairs of the Government of Spain will be determined (55).

### 2.29 Screening for eating disorders

To evaluate disordered eating, the modified SCOFF (mSCOFF) questionnaire will be used. This screening tool consists of five straightforward questions and can be conveniently incorporated into a routine check-up. It has proven to be reliable and valid in its assessment of eating disorders among children and adolescents with T1DM (56).

### 2.30 App usability

We will use the Spanish Version of the User Version of the Mobile Application Rating Scale (uMARS) (57), that will serve as a comprehensive and objective measure of app usability, consisting of 20 items. Each item is assessed using a 5-point scale, ranging from 1 (inadequate) to 5 (excellent). The uMARS will be structured into four subscales: engagement, functionality, aesthetics, and information quality. Subscale scores will be calculated as the average of their respective items, and the mean of these subscale scores will provide an overall app quality score.

### 2.31 Participant timeline

Eligible and consenting participants will complete a baseline assessment. The intervention group will then be allocated to a 24-weeks exercise training program using the Diactive-1 App. The control group will continue standard care. Outcomes will be assessed at baseline (T0), 12-week (T1) and after the 24-week intervention period (T2) (**Table 2**).

**Table 2:** Schedule depicting the enrollment and interventions for the Diactive-1 Study in accordance with the SPIRIT 2013 guidelines ^26^.

TIMEPOINT	STUDY PERIOD				
	Enrolment	Allocation		Post-allocation	
	*-t_1_*	0	*Baseline* *(T0)*	*T_1_* *_(12 weeks)_*	*T_2_* *_(24 weeks)_*
ENROLMENT:					
Eligibility screen	X				
Informed consent	X				
Allocation		X			
INTERVENTIONS:					
*Diactive-1*		X	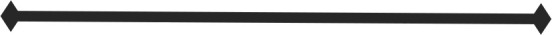		
*Waiting-list control group*		X	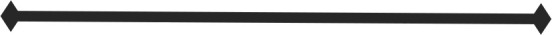		
ASSESSMENTS:					
*Daily insulin dosage requirement*			X	X	X
*Glycemic control (time in range, glucose coefficient of variation, number of hypoglycemic and hyperglycemic events)*			X		X
*Cardiovascular biomarkers (of fasting glucose, glycated hemoglobin, triglycerides, high-density lipoprotein cholesterol, low-density lipoprotein cholesterol, apolipoproteins, alanine aminotransferase, and aspartate aminotransferase)*			X		X
*Anthropometric parameters (body weight, standing and sitting height)*			X		X
*Sexual maturation and peak height velocity*			X		X
*Body composition parameters (total body fat, lean mass, subcutaneous and visceral adiposity, bone mineral content and density)*			X	X	X
*Heart rate variability*			X		X
*Vascular function*			X		X
Physical fitness components (cardiorespiratory and muscular fitness)*			X	X	X
Physical activity, sedentary time, and sleep duration by accelerometers and questionnaires *			X	X	X
*Sleep quality*			X		X
*Inadvertent hypoglycemia*			X		X
*Psychological assessments (Health-Related Quality of Life and subjective well-being)*			X		X
*Diet (adherence to the Mediterranean diet and food consumption)*			X		X
*Eating disorders*			X		X
*App usability*					X

* It will only evaluate handgrip strength and accelerometer-based physical activity at 12-week mark. T, represents a time-point.

### 2.32 Sample size and recruitment

At least 52 children and adolescents of both sexes, aged between 8 and 18 years old, will be recruited from the Pediatric Endocrinology Unit at University Hospital of Navarra (Pamplona, Spain). This sample size was determined based on the results of a previous meta-analysis (12), which suggested an expected effect size on daily insulin dose requirements of 0.81. Using the G*Power software (58) and considering a power of 0.80, a significance level of 0.05 and accounting for a dropout of 15% (59, 60), a minimum of 26 children and adolescents per group is required.

### 2.33 Randomization, allocation concealment, and blinding

Participants meeting eligibility criteria will be randomly assigned to one of two groups in a 1:1 ratio using block randomization with a computer-generated schedule (Research Randomizer V.4). This will ensure equal group sizes. Randomization will continue until a predetermined number of participants are assigned. The allocation code will be kept confidential at Navarrabiomed until final analysis. To ensure blinding, each study group will be assigned an alpha-numeric code. Researchers will receive the code for their assigned group. Data analysts will not have access to the code until completing the coded intervention analysis. Due to the nature of the study, patients in the Diactive-1 App group will not be blinded.

### 2.34 Procedure for unblinding if needed.

This study is an unblinded, practice-level intervention.

### 2.35 Data collection methods and management

Researchers will be responsible for finalizing the study protocol and maintaining regular communication through phone calls, emails, and meetings. Weekly meetings will also be held for investigators and pediatric staff to discuss progress and updates.

The provided information will be documented in the database using individualized study codes assigned to each participant. This data will be securely stored on a computer that requires a password for access. Only the data manager, who operates independently and without conflicting interests, will have permission to access and retrieve the data. Due to the low level of risk associated with the study, there is no requirement for a Data Monitoring Committee. However, any significant modifications to the study protocol will be promptly communicated and updated on both the Clinical Trial Registry and the publication journal. Within a maximum timeframe of three years from the collection of the end-line assessment at the 24-week mark, a fully anonymized dataset will be submitted to an appropriate data archive for sharing purposes.

### 2.36 Statistical methods

The quantitative variables will have their mean (M) and standard deviation (SD) provided, whereas the qualitative variables will include frequencies (n) and percentages (%). To assess data normality visual inspection of Q-Q plots and Shapiro-Wilk test will be used. For the homogeneity of variances, Levene’s test will be used. Subsequently, for two-group comparisons, either Student’s t-test or Mann–Whitney U test will be used based on adherence to the normality assumption.

Associations among qualitative variables will be examined using Pearson’s chi-square (χ²) test. For quantitative variables, the association will be tested using Spearman’s rank correlation coefficient (ρ) or Pearson’s correlation coefficient (r), depending on the assumption of normality. Initial analyses will establish frequency, range, variability, and distribution patterns of each variable, guiding the choice of the most appropriate statistical test for comparisons.

Given the experimental design of this RCT involving two data collections—baseline (t0 = 0 weeks) and post-intervention (t1, t2 = 12 and 24 weeks)—in both intervention and control groups, a comparative analysis will be conducted to identify intergroup differences. Multilevel mixed-effects regression models with repeated measures will be used to evaluate the intervention effect for each dependent variable. Multivariate analyses will account for autocorrelation between repeated measures.

For data analysis, both intention-to-treat (ITT) and per-protocol (PP) approaches will be employed. ITT measures the impact of intervention assignment, while PP analysis gauges the effect of intervention receipt. Statistical analyses will be performed using Stata software (version 17.0) (StataCorp, College Station, TX, USA), the statistical software R (Version 4.1.1) (R Core Team, Vienna, Austria), and RStudio (Version 2021.09.2) (Posit, Boston, MA, USA). Statistical significance will be determined by a p-value ≤ 0.05.

In situations where exercise sessions are missing, the analysis of outcomes will incorporate a dose-response approach. This approach will consider potential variations in outcome measurements based on the degree of exposure to the intervention. Additionally, depending on the nature of the missing data for both primary and secondary outcome measures, a range of techniques like multiple imputation, listwise deletion, or specific analytical methods will be employed. These measures aim to mitigate the impact of missing data, thereby minimizing any potential reduction in the generalizability of the collected data (61).

Subgroup analyses will be carried out to ascertain whether exercise is more or less effective in reducing secondary outcomes compared to the waiting-list control group. These subgroup analyses will adhere to the same methodology as the primary analysis, including both the primary analysis variables and their interaction with the experimental condition.

### 2.37 Data monitoring

The team will oversee and document any unfavorable incidents, ensuring that severe adverse events are promptly reported to the designated committee, following their recommendations. There won’t be a requirement for a formal data monitoring committee in this particular RCT. The need for a data monitoring committee was not deemed necessary given the low-risk nature of this intervention.

### 2.38 Adverse event reporting and harms

Serious adverse events associated with the intervention will be reported to the Pediatric Endocrinology Unit at the ethics committees from the University Hospital of Navarra (Pamplona, Navarra).

### 2.39 Frequency and plans for auditing trial conduct

As detailed in this protocol, the team will conduct weekly monitoring of all RCT aspects. This encompasses ensuring adherence to the protocol, maintaining ethical and governance standards, overseeing database management, assessing outcomes, conducting research staff training, and regularly reporting on informed consent.

## 3 Discussion

This 24-week study aims to explore reductions in daily insulin dosage per kilogram of weight through the use of the Diactive-1 App in children and adolescents with T1DM. Including the assessment of daily insulin dosage in an intervention for this population is vital for various reasons. Firstly, it enables the monitoring of glycemic control, facilitating adjustments in insulin dosage to prevent hypo- and hyperglycemia (10). Secondly, the regular review and adjustment of the daily insulin dose are necessary, considering factors such as age, sex, BMI, pubertal status, and mode of therapy, to ensure optimal glucose control (62). Therefore, regular assessment and adjustment of the daily insulin dose are essential for personalized treatment (63).

Additionally, the study will assess glucose control, physical activity levels, body composition, app usability, and other health outcomes. Previous investigations have demonstrated that exercise training exerts a positive impact on metabolic and psychological health in children and adolescents with T1DM (12, 64). The evidence also indicates that smartphone-based intervention may be a promising strategy to increase physical activity in children and adolescents (65). Therefore, while exercise interventions offer positive effects on physiological and biochemical outcomes, including glycemic control and body composition, personalized approaches to exercise promotion and meticulous management of insulin doses are crucial for this demographic (66). Since it has previously demonstrated promising results with just two weekly exercise sessions, the use of strength training in this population is also innovative (13).

The integration of technology is central to this study, demonstrating the potential of apps and digital platforms to transform diabetes care. This shift from traditional methods enables clinicians to explore integrating mobile apps into care. A recent meta-analysis in young patients with T1DM revealed a non-significant trend of reduced HbA1c levels from the beginning to the end of the study when using smartphone apps, and this reduction did not lead to an increase in hypoglycemia (67). While not statistically significant, this suggests apps could provide consistent monitoring and education to manage T1DM with minimal intrusion. The goals of the studies included in this meta-analysis are to improve glycemic control through diverse strategies, such as promoting glucose monitoring, facilitating data collection, coaching individuals with diabetes, providing guidance on healthy nutrition and medication dosing, and supporting lifestyle modifications. Building on this previous research and leveraging emerging tech for T1DM, the Diactive-1 Study is expected to provide a personalized, user-friendly non-pharmacological intervention for managing T1DM through the prescription of physical exercise. Additionally, the study incorporates education on insulin and carbohydrate management in the context of this exercise, enhancing the comprehensive approach to T1DM care.

The study’s recognition of resistance exercise as a therapeutic strategy reflects a paradigm shift in managing T1DM (10). It underscores the potential for exercise to become an integral component of the overall treatment plan, extending beyond purely physical benefits. By encouraging clinicians to tailor exercise prescriptions to each patient’s unique needs, age, capabilities, and glucose requirements, this approach elevates exercise to a personalized therapeutic strategy that can significantly enhance holistic T1DM management. The study also underscores the significance of tailored education for patients regarding glycemic control and exercise. This emphasis on empowering young patients can help cultivate self-confidence, enhance monitoring capabilities, and develop decision-making skills, enabling them to proactively manage their condition and daily activities. Educational strategies that prioritize self-management empower clinicians to effectively instill a sense of ownership and agency in children and adolescents.

In conclusion, smartphone apps for diabetes management have shown encouraging trends in improving glycemic control, indicating the potential of mobile apps as valuable tools for effective management. Aligning with this evolving landscape, the Diactive-1 study capitalizes on this trend through a groundbreaking mHealth App exploring potential benefits for various aspects of T1DM management, including personalized physical exercise. The insights from this study could play a pivotal role in shaping health promotion and prevention initiatives that leverage technology innovations to significantly benefit individuals navigating T1DM challenges.

## Data availability statement

The original contributions presented in the study are included in the article/**Supplementary Material**. Further inquiries can be directed to the corresponding author.

## Ethics statement

The studies involving humans were approved by University Hospital of Navarra Research Board (PI_2020/140). The studies were conducted in accordance with the local legislation and institutional requirements. Written informed consent for participation in this study was provided by the participants’ legal guardians/next of kin. Written informed consent was obtained from the individual(s), and minor(s)’ legal guardian/next of kin, for the publication of any potentially identifiable images or data included in this article.

## Author contributions

IH: Formal analysis, Visualization, Writing – original draft. JM: Resources, Writing – review & editing. JL: Conceptualization, Formal analysis, Funding acquisition, Investigation, Writing – review & editing. NH: Project administration, Writing – review & editing. MC: Funding acquisition, Methodology, Resources, Writing – review & editing. SB: Funding acquisition, Resources, Writing – review & editing. ES: Supervision, Writing – review & editing. MI: Conceptualization, Investigation, Writing – review & editing. YE: Funding acquisition, Visualization, Writing – review & editing. AG-H Conceptualization, Data curation, Formal analysis, Funding acquisition, Investigation, Methodology, Project administration, Software, Supervision, Writing – original draft, Writing – review & editing.
