# Supplementary material for: Comprehensive management of children and adolescents with type 1 diabetes mellitus through personalized physical exercise and education using an mHealth system: The Diactive-1 study protocol
Source: Front Endocrinol (Lausanne). 2024 Feb 6;15:1354734. doi: 10.3389/fendo.2024.1354734 (PMC10877052; doi:10.3389/fendo.2024.1354734)
Supplement: Supplementary file 1 [file Table_1.docx]

Supplementary Material

# Supplementary Tables

| Supplementary Table 1. Example of resistance training progression for children and adolescents with low fitness using aquaball or elastic band | | | | |
| --- | --- | --- | --- | --- |
|  | **Execution time of the exercise (seconds)** | **Rest between series (seconds)** | **Rest between exercises (seconds)** | **Exercises per session** |
| *Children (8-12 years old)* | | | | |
| Low Fitness + upper limb | 20-40 | 30 | 60 | 4 |
| Low Fitness + lower limb | 20-40 | 30 | 60 | 4 |
| Low fitness + core | 20-40 | 30 | 60 | 4 |
| *Adolescents (13-18 years old)* | | | | |
| Low Fitness + upper limb | 20-40 | 30 | 60 | 4 |
| Low Fitness + lower limb | 20-40 | 30 | 60 | 4 |
| Low fitness + core | 20-40 | 30 | 60 | 4 |

| Supplementary Table 2. Example of lower limb training progression for children and adolescents with low fitness using aquaball (kg) or elastic band | | | | | |
| --- | --- | --- | --- | --- | --- |
| **Series** | **Repetitions** | **Load** | | **Execution time (seconds)** | **Total time (minutes)** |
|  |  | **Weight (Kg)** | **Color band** |  |  |
| *Children (8-12 years old)* | | | | | |
| 3 | 6 | 5 | Green | 20 | 11 |
| 3 | 9 | 5 | Green | 30 | 13 |
| 3 | 12 | 5 | Green | 40 | 15 |
| 3 | 9 | 6 | Green | 30 | 13 |
| 3 | 12 | 6 | Green | 40 | 15 |
| 3 | 9 | 6.5 | Green | 30 | 13 |
| 3 | 12 | 6.5 | Green | 40 | 15 |
| 3 | 9 | 7 | Green | 30 | 13 |
| 3 | 12 | 7 | Green | 40 | 15 |
| 4 | 9 | 7.5 | Green | 30 | 17 |
| 4 | 12 | 7.5 | Green | 40 | 19 |
| *Adolescents (13-18 years old)* | | | | | |
| 3 | 6 | 8 | Green | 20 | 11 |
| 3 | 9 | 8 | Green | 30 | 13 |
| 3 | 12 | 8 | Green | 40 | 15 |
| 3 | 9 | 10 | Green | 30 | 13 |
| 3 | 12 | 10 | Green | 40 | 15 |
| 3 | 9 | 11 | Green | 30 | 13 |
| 3 | 12 | 11 | Green | 40 | 15 |
| 3 | 9 | 12 | Green | 30 | 13 |
| 3 | 12 | 12 | Green | 40 | 15 |
| 4 | 9 | 13.5 | Green | 30 | 17 |
| 4 | 12 | 13.5 | Green | 40 | 19 |

| Supplementary Table 3. Example of resistance training progression for children and adolescents with medium fitness using aquaball (kg) or elastic band | | | | |
| --- | --- | --- | --- | --- |
|  | **Execution time of the exercise (seconds)** | **Rest between series (seconds)** | **Rest between exercises (seconds)** | **Exercises per session** |
| *Children (8-12 years old)* | | | | |
| Medium Fitness + upper limb | 20-40 | 45 | 60 | 5 |
| Medium Fitness + lower limb | 20-40 | 45 | 60 | 5 |
| Medium Fitness + core | 20-40 | 45 | 60 | 5 |
| *Adolescents (13-18 years old)* | | | | |
| Medium Fitness + upper limb | 20-40 | 45 | 60 | 5 |
| Medium Fitness + lower limb | 20-40 | 45 | 60 | 5 |
| Medium Fitness + core | 20-40 | 45 | 60 | 5 |

| Supplementary Table 4. Example of upper limb training progression for children and adolescents with medium fitness using aquaball (kg) or elastic band | | | | | |
| --- | --- | --- | --- | --- | --- |
| **Series** | **Repetitions** | **Load** | | **Execution time (seconds)** | **Total time (minutes)** |
|  |  | **Weight (Kg)** | **Color band** |  |  |
| *Children (8-12 years old)* | | | | | |
| 3 | 6 | 4 | Orange | 20 | 16 |
| 3 | 9 | 4 | Orange | 30 | 19 |
| 3 | 12 | 4 | Orange | 40 | 21 |
| 3 | 9 | 4.5 | Orange | 30 | 19 |
| 3 | 12 | 4.5 | Orange | 40 | 21 |
| 3 | 9 | 5 | Orange | 30 | 19 |
| 3 | 12 | 5 | Orange | 40 | 21 |
| 3 | 9 | 5.5 | Orange | 30 | 19 |
| 3 | 12 | 5.5 | Orange | 40 | 21 |
| 4 | 9 | 5.5 | Orange | 30 | 25 |
| 4 | 12 | 5.5 | Orange | 40 | 28 |
| *Adolescents (13-18 years old)* | | | | | |
| 3 | 6 | 6 | Orange | 20 | 16 |
| 3 | 9 | 6 | Orange | 30 | 19 |
| 3 | 12 | 6 | Orange | 40 | 21 |
| 3 | 9 | 6.5 | Orange | 30 | 19 |
| 3 | 12 | 6.5 | Orange | 40 | 21 |
| 3 | 9 | 7 | Orange | 30 | 19 |
| 3 | 12 | 7 | Orange | 40 | 21 |
| 3 | 9 | 7.5 | Orange | 30 | 19 |
| 3 | 12 | 7.5 | Orange | 40 | 21 |
| 4 | 9 | 7.5 | Orange | 30 | 25 |
| 4 | 12 | 7.5 | Orange | 40 | 28 |

| Supplementary Table 5. Example of resistance training progression for children (8-12 years old) and adolescents (13-18 years old) with high fitness and without equipment | | | | |
| --- | --- | --- | --- | --- |
|  | **Execution time of the exercise (seconds)** | **Rest between series (seconds)** | **Rest between exercises (seconds)** | **Exercises per session** |
| High Fitness + upper limb | 20-40 | 60 | 75 | 5 |
| High Fitness + lower limb | 20-40 | 60 | 75 | 5 |
| High fitness + core | 20-40 | 60 | 75 | 5 |

| Supplementary Table 6. Example of core training progression for children (8-12 years old) and adolescents (13-18 years old) with high fitness and without equipment | | | | |
| --- | --- | --- | --- | --- |
| **Series** | **Repetitions** | **Load** | **Execution time (seconds)** | **Total time (minutes)** |
| 3 | 6 | Body weight | 20 | 20 |
| 4 | 6 | Body weight | 20 | 26 |
| 3 | 9 | Body weight | 30 | 22 |
| 3 | 12 | Body weight | 40 | 25 |
| 4 | 9 | Body weight | 30 | 30 |
| 4 | 12 | Body weight | 40 | 33 |

| Supplementary Table 7. Example of lower limb training progression for children (8-12 years old) and adolescents (13-18 years old) with high fitness and without equipment | | | | |
| --- | --- | --- | --- | --- |
| **Series** | **Repetitions** | **Load** | **Execution time (seconds)** | **Total time (minutes)** |
| 3 | 6 | Body weight | 20 | 20 |
| 4 | 6 | Body weight | 20 | 26 |
| 3 | 9 | Body weight | 30 | 22 |
| 3 | 12 | Body weight | 40 | 25 |
| 4 | 9 | Body weight | 30 | 30 |
| 4 | 12 | Body weight | 40 | 33 |

| Supplementary Table 8. Example of resistance training progression for children (8-12 years old) and adolescents (13-18 years old) with a partner | | | | |
| --- | --- | --- | --- | --- |
|  | **Execution time of the exercise (seconds)** | **Rest between series (seconds)** | **Rest between exercises (seconds)** | **Quantity of exercises per session** |
| Low Fitness | 20-40 | 45 | 60 | 5 |
| Medium Fitness | 20-40 | 45 | 60 | 5 |
| High Fitness | 20-40 | 45 | 60 | 5 |

| Supplementary Table 9. Example of full body training progression for children and adolescents with a partner. | | | | |
| --- | --- | --- | --- | --- |
| **Series** | **Repetitions** | **Load** | **Execution time (seconds)** | **Total time (minutes)** |
| 3 | 6 | Body weight | 20 | 16 |
| 4 | 6 | Body weight | 20 | 21 |
| 3 | 9 | Body weight | 30 | 19 |
| 4 | 9 | Body weight | 30 | 25 |
| 3 | 12 | Body weight | 40 | 21 |
| 4 | 12 | Body weight | 40 | 28 |

| Supplementary Table 10. Example of an aerobic exercise progression to aid in lowering blood glucose levels in children and adolescents both with a partner and without a partner. | | | | | |
| --- | --- | --- | --- | --- | --- |
| **Fitness level** | **Execution time (seconds)** | **Rest between series** | **Rest between exercises** | **Quantity of exercises per session** | **Total time (minutes)** |
| All fitness levels | 60 | 10 | 45 | 6 | 20 |
